# Association between lipid accumulation product and endometriosis: A cross-sectional study from NHANES 1999–2006

**DOI:** 10.1371/journal.pone.0323932

**Published:** 2025-05-15

**Authors:** Jing Zhe, Yanxing Cai, Yakun Bi

**Affiliations:** 1 Center for Reproductive Medicine, Department of Obstetrics and Gynecology, Guiyang Maternal and Child Health Care Hospital, Guiyang Children’s Hospital, Guiyang, China; 2 Department of Clinical Laboratory, Guiyang Maternal and Child Health Care Hospital, Guiyang Children’s Hospital, Guiyang, China; 3 Science and Technology Management Center, Guiyang Maternal and Child Health Care Hospital, Guiyang Children’s Hospital, Guiyang, China; Noorda College of Osteopathic Medicine, UNITED STATES OF AMERICA

## Abstract

The association of lipid accumulation product (LAP) and the likelihood of endometriosis prevalence has not been previously mentioned. The research aimed to assess the possible potential association between LAP and endometriosis in nationwide research. This cross-sectional analysis was conducted on 2,216 participants selected from the National Health and Nutrition Examination Survey (NHANES) in the 1999–2006 cycles. Logistic regression and stratified analysis by age, race, level of education, BMI, marital status, PIR, glycohemoglobin, drinking, and smoking status were used to analyze the association of the LAP index and odds of endometriosis prevalence. Moreover, smoothed curve fitting was used to evaluate the relevancy of LAP and endometriosis. The multivariate logistic regression model showed a positive association between ln LAP and endometriosis. This trend remained after a full adjustment (odds ratio = 1.37, 95% confidence interval:1.08–1.75, *P* = 0.010). Compared to the minimum ln LAP quartile, participants in the highest ln LAP had a 93% higher chance of endometriosis incidence (odds ratio = 1.93, 95% confidence interval: 1.08–3.46, *P* = 0.027). After conducting subgroup analysis and interaction testing, it was found that this positive association was most prominent among women aged 35 years and above and participants with glycohemoglobin≥6%. This nationwide study suggested that an elevated ln LAP was related to an increased endometriosis prevalence. Therefore, LAP may be a valuable tool for predicting the occurrence of endometriosis. Follow-up studies are critical to assess the association between LAP and odds of endometriosis prevalence and explain the potential mechanisms of this relationship.

## Introduction

Endometriosis is a common, gynecologic condition defined as the formation of ectopic endometrial tissue in non-uterine regions [1]. According to reports, approximately 5% to 15% of reproductive-aged women suffer from this chronic systemic disease [2–4]. Endometriosis can also lead to female infertility in addition to causing severe pain and irregular menstrual cycles [2]. Women with endometriosis can have negative impacts on interpersonal relationships, quality of life, and work efficiency of individuals and families [5]. However, the main methods for treating endometriosis currently include drug therapy and surgical resection, and their therapeutic quality of life effects are limited [6]. Further research is urgently needed to identify more effective markers for evaluation, prevention, diagnosis, and management strategies.

Although there are several factors, such as inflammation, hormones, metabolism, and immunology, that have been implicated in endometriosis, the specific pathological mechanism of endometriosis is still not fully understood [6]. Recent studies have shown that metabolic abnormalities are key to the occurrence and development of endometriosis [7,8]. Especially, women diagnosed with endometriosis have been recognized to have abnormal lipid and glucose metabolism [9–11].

Lipid accumulation product (LAP) is an index that combines fasting triglyceride (TG) and waist circumference (WC) [12]. Kahn studied LAP in 2005 and found it to be a reliable index of lipid accumulation [12]. Currently, studies have provided evidence that LAP can be used as a potential marker for metabolic syndrome (MetS), type 2 diabetes, insulin resistance, and cardiovascular diseases [13–16]. In addition, the LAP index is not affected by muscle mass and is suitable for the elderly and people with higher muscular density [10].

So far, there has been little exploration of the relationship between lipid accumulation products and endometriosis. Therefore, the study aimed to investigate the association between LAP and endometriosis using data from NHANES in the 1999–2006 cycles. The overall objective is to extend a new perspective to the precaution and therapy of endometriosis.

## Materials and methods

### Data sources

NHANES is a project that began in the early 1960s to provide data for this study. NHANES is a research program that combines physical examinations and interviews to estimate the nutrition and overall health of the US population. Every year, it applies a multi-stage sampling design to select a sample nationwide of approximately 5,000 individuals [17]. The NHANES survey plan was developed by the National Research Ethics Review Committee, and study individuals gave informed permission [18].

### Study population

**Fig 1:**
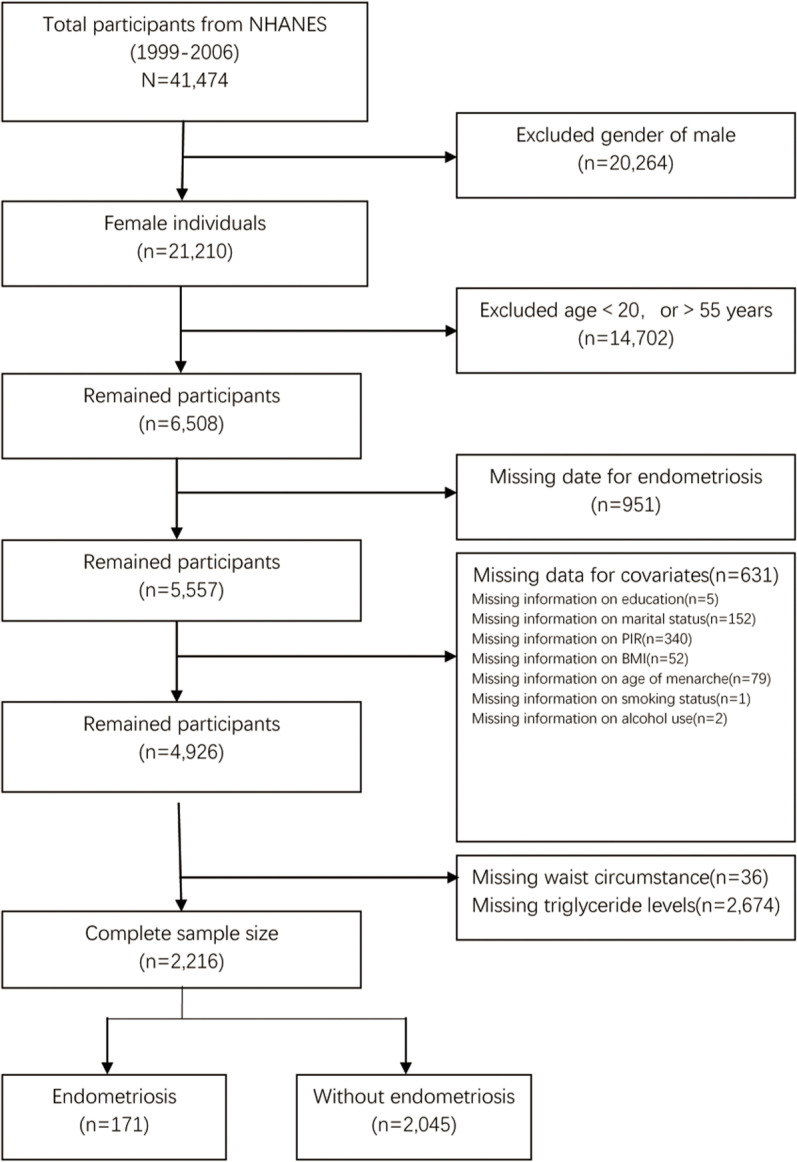
Flowchart of the sample selection from NHANES 1999–2006.

Relevant data came from the NHANES cycle from 1999 to 2006. 41,474 people participated in this cross-sectional study. Through this extensive examination and analysis of NHANES, we excluded male individuals (n = 20,264) and female participants aged 20 and younger and 55 and older (n = 6,508). Then we excluded individuals without complete data on endometriosis. Additionally, participants with missing information on educational levels (n = 5), marital status (n = 152), the ratio of family income to poverty (n = 340), BMI (n = 52), age when first menstrual period occurred (n = 79), smoking status (n = 1) and alcohol use (n = 2) were excluded. Subsequently, participants with incomplete waist circumstance (n = 36) and triglyceride levels (n = 2,674) data were also excluded from the study. Ultimately, 2,216 participants were enrolled (Fig 1).

### Assessment of endometriosis (dependent variable)

The main outcome was self-reported endometriosis from the Reproductive Health Questionnaire (variable name in the questionnaire: RHQ360). Participants were asked, “Has a doctor or other health professional ever told you that you have endometriosis?” Those who answered “Yes” were considered to have endometriosis.

### LAP index calculation (independent variable)

Serum triglyceride levels (TG, mmol/L) and waist circumference (WC, cm) are variables in the gender-specific LAP calculation. The formula is [WC (cm)-58] × [TG (mmol/l)] for women [19–20]. Ln LAP was a natural log transformation of the LAP index. All participants’ LAP index and ln LAP were subsequently calculated using these values, and then they were divided into quartiles (Q1-Q4) for analysis.

### Covariates

Based on previous literature [21,22] and clinical experience, potential covariates included age (years), race, educational level, marital status, body mass index (BMI), early menstruation before the age of 11, glycohemoglobin, poverty income ratio (PIR), smoking, and alcohol use. The level of education was reported as below high school, high school, and above high school [23]. BMI was categorized as BMI ＜ 25 kg/m^2^, 25 kg/m^2^ ≤ BMI ＜ 30 kg/m^2^, and BMI ≥ 30 kg/m^2^. Age at menarche was collected by using ‘RHQ10’ questionnaires. Participants were asked, “Age when first menstrual period occurred” [24]. PIR was assessed as PIR ＜ 1.35, 1.35 ≤ PIR ＜ 3.0, and PIR ≥ 3.0 by the degree of poverty [25]. Glycohemoglobin was collected in laboratory data(＜6% and ≥6%). Other covariates relevant to health, such as alcohol consumption and smoking status, were analyzed in the MEC. The smoking situation was divided into yes or no. Participants with a minimum of 100 cigarettes throughout their lifespan are defined as smokers. Alcohol use was classified as never, former, or current. Participants with no drinking alcohol were defined as not having consumed 12 alcohol drinks/lifetime.

### Statistical analysis

This study’s data analysis utilized package R version 3.4.3 and EmpowerStats software. The individuals were firstly divided into two groups: one for endometriosis and the other for control; these groups were referred to as the “Endometriosis” and “Control” groups, respectively. There was a 0.05 significance limit. To more fully indicate the significance level of the results, use asterisks (*) to mark them. Significant levels were 0.05 (*), 0.01 (**), and 0.001 (***). The grouping variable was shown as percentages and continuous variable data was shown as Mean±SD (standard deviations). A natural log transformation, identified as ‘ln LAP’ was utilized to convert the non-normal distribution of LAP into a normal distribution. Two approaches were applied in the study to evaluate group differences: weighted linear regression was performed for continuous variables, and the weighted chi-square test was used for categorical data. Multivariable logistic regression models were used to assess the association between ln LAP and endometriosis. Model 1 (the crude model) had no adjustment. Model 2 (the minimally adjusted model) was adjusted for age and race. Model 3 (the fully adjusted model) was adjusted for the variables in Model 2 plus level of education, marital status, BMI, PIR, early menstruation before the age of 11, glycohemoglobin, alcohol use, and smoking status. Then ln LAP was regarded as a categorical variable by quartile. To investigate the potential association between LAP and odds of endometriosis incidence, a smoothed curve fitting was employed. Subgroup stratified by age and race, educational level, BMI, marital status, PIR, early menstruation before the age of 11, glycohemoglobin, drinking, and smoking status were also utilized. The logistic regression and subgroup analysis are expressed as odds ratios (OR) and 95% confidence intervals (CI).

## Results

### Basic characteristics

The baseline data of 2,216 participants in this study were summarized in Table 1. Among these, 171 (7.72%) were diagnosed with endometriosis, whereas 2,045 cases were not. During the whole study process, the age range of the women participating in this study was 20–54 years. In contrast to 36.91 ± 10.02 years in those without endometriosis, patients indicated a higher incidence in elderly people(*P* < 0.001). Family income to poverty ratio (PIR), LAP, race, educational level, marital status, and smoking status all showed a significant relationship with endometriosis. More precisely, those who were non-Hispanic White, completed at least high school, were in smoking condition, had higher PIR, and had a status of married or living with partners were more frequent in those with this condition.

**Table 1:** Baseline characteristics of participants with and without endometriosis.

	Total (N = 2,216)	Non-endometriosis (n = 2,045)	Endometriosis (n = 171)	*P*-value
Age(years)	37.25 ± 9.90	36.91 ± 10.02	40.17 ± 8.27	<0.001***
PIR	3.01 ± 1.64	2.99 ± 1.64	3.25 ± 1.64	0.021*
BMI (kg/m^2^)	28.03 ± 7.08	27.98 ± 7.12	28.47 ± 6.69	0.316
Glycohemoglobin(%)	5.28 ± 0.75	5.28 ± 0.75	5.31 ± 0.79	0.457
LAP	51.83 ± 58.31	49.39 ± 50.41	72.99 ± 101.80	<0.001***
ln LAP	3.56 ± 0.88	3.53 ± 0.87	3.83 ± 0.91	<0.001***
Race, %				<0.001***
Non-Hispanic White	69.57	67.77	85.21	
Non-Hispanic Black	12.16	12.67	7.66	
Mexican American	7.38	8.07	1.43	
Other race	10.89	11.49	5.70	
Educational level, %				0.004**
Below high school	15.16	15.75	10.01	
High school	23.13	22.26	30.75	
Above high school	61.71	61.99	59.24	
Marital status, %				0.001**
Married/living with partners	67.56	66.68	75.23	
Widowed/divorced/separated	15.00	14.84	16.37	
Never married	17.44	18.48	8.40	
Age of menarche(years)				0.544
＜11	0.40	0.43	99.57	
≥11	99.60	0.16	99.84	
Smoking status, %				<0.001***
Yes	42.60	40.82	58.08	
No	57.40	59.18	41.92	
Alcohol use, %				0.916
Never	20.57	20.55	20.80	
Former	7.91	7.83	8.57	
Now	71.52	71.62	70.63	

Mean ±SD for continuous variables; *P* value was calculated by the weighted linear regression model. % for categorical variables: *P* value was calculated by weighted chi-square test. PIR, family income to poverty ratio; BMI, Body mass index.

Nevertheless, there was no statistically significant association between the chance of endometriosis prevalence and BMI, with a *P*-value above 0.05. The use of alcohol and the age of menarche represented similar trends. Furthermore, there is a significant difference in LAP between the two groups.

#### Relationships between ln LAP and endometriosis.

Logistic regression models were investigated in Table 2. The ln LAP and endometriosis have been demonstrated to have a strong positive association in the crude model (OR=1.23, 95% CI:1.03–1.47, *P* = 0.023). After a full adjustment in Model 2, this positive association remained (OR=1.37, 95% CI:1.08–1.75, *P* = 0.010), suggesting a 0.37-fold raise in the odds of endometriosis incidence for every unit elevation in the ln LAP.

**Table 2:** Association between LAP index and endometriosis prevalence risk.

	Crude Model OR (95%CI), *P*-value	Model 1 OR (95%CI), *P*-value	Model 2 OR (95%CI), *P*-value
ln LAP	1.23 (1.03, 1.47) 0.023*	1.30 (1.08, 1.56) 0.006**	1.37 (1.08, 1.75) 0.010*
ln LAP index Quartile			
Tertile 1	1.0	1.0	1.0
Tertile 2	1.22 (0.76, 1.96) 0.403	1.25 (0.77, 2.02) 0.367	1.30 (0.79, 2.14) 0.298
Tertile 3	1.35 (0.85, 2.15) 0.200	1.43 (0.89, 2.29) 0.142	1.50 (0.88, 2.56) 0.133
Tertile 4	1.55 (0.99, 2.43) 0.057	1.76 (1.11, 2.79) 0.016	1.93 (1.08, 3.46) 0.027*

The crude model adjusts for: none.

Model 1 adjusts for age and race.

Model 2 adjusts for age, race, educational level, marital status, BMI, PIR, early menstruation before the age of 11, glycohemoglobin, smoking status, and alcohol use.

**Fig 2:**
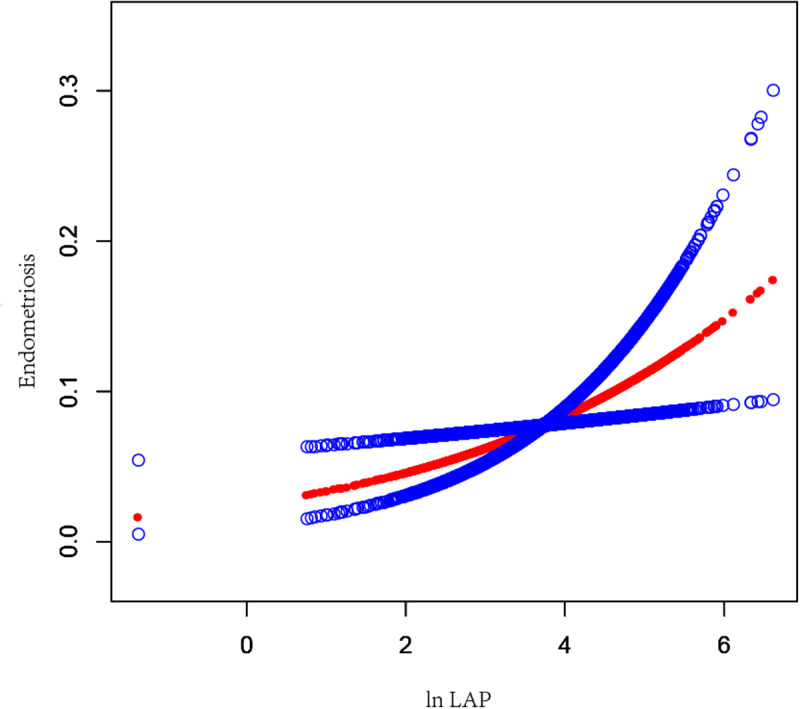
Ln LAP and infertility have a positive linear connection. Ln LAP was a natural log transformation of the LAP index. The solid red line represents the smooth curve fit between variables. Blue bands represent the 95% confidence interval from the fit.

**Fig 3:**
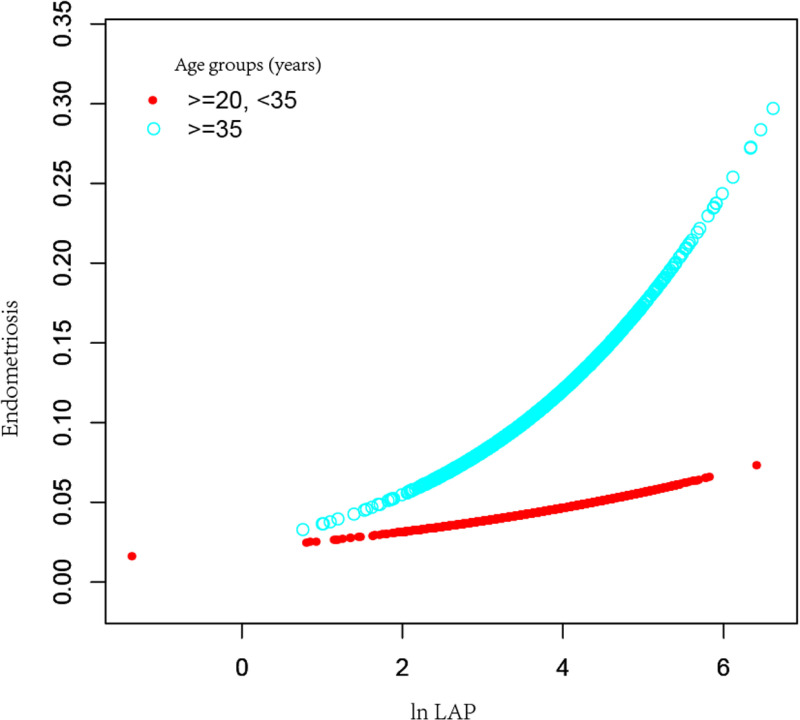
The association between ln LAP and endometriosis prevalence stratified by age groups. Ln LAP was a natural log transformation of the LAP index. The red line represents the population aged 20 to 35, while the blue line represents women over 35 years old.

**Fig 4:**
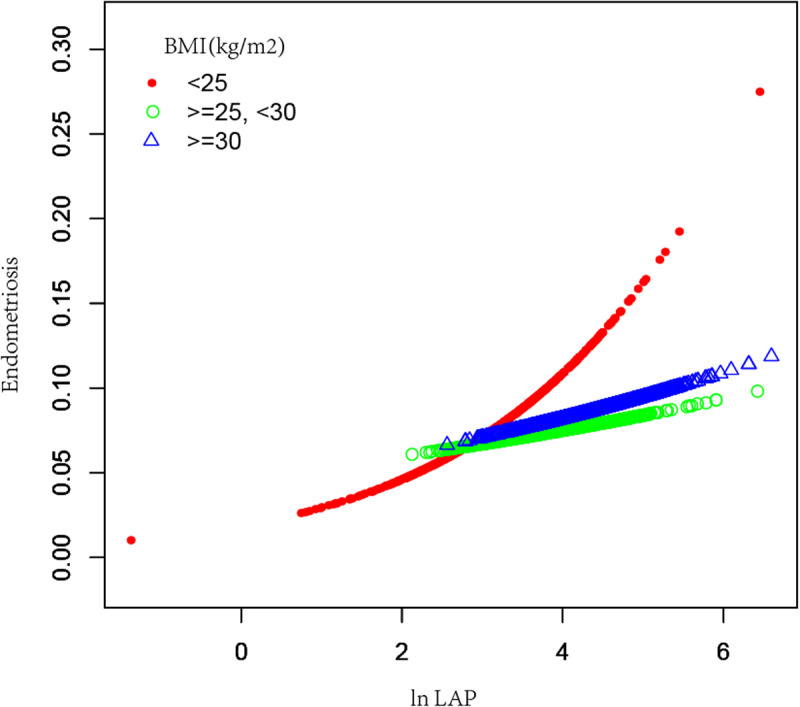
The association between ln LAP and endometriosis stratified by BMI groups. Ln LAP was a natural log transformation of the LAP index. The red, green, and blue lines are used to classify the population with BMI ＜ 25 kg/m^2^, 25-30 kg/m^2^ and ≥30 kg/m^2^.

**Fig 5:**
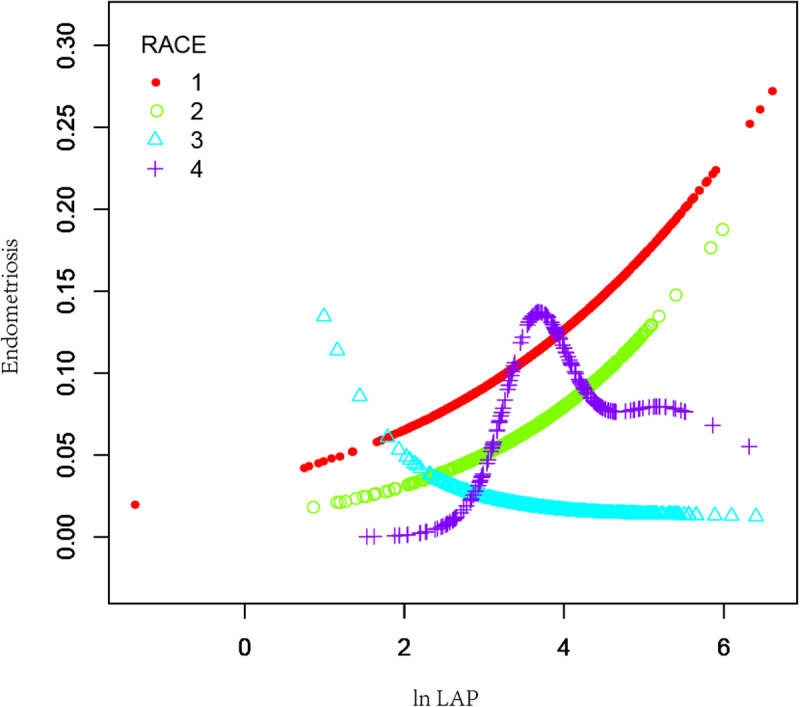
The association between ln LAP and endometriosis stratified by race. Ln LAP was a natural log transformation of the LAP index.1: Non-Hispanic White; 2. Non-Hispanic Black; 3. Mexican American; 4: Other Race.

To further conduct the association between ln LAP and endometriosis, ln LAP was divided into four tertiles. Compared to the Tertile-1- ln LAP in Model 2, the OR for the Tertile-4-ln LAP was 1.93(95% CI:1.08–3.46, *P* = 0.027), suggesting a substantial 0.93-fold increase in the chance of endometriosis prevalence in Tertile 4. However, Tertile 1 and Tertile 2 did not differ statistically significantly. As shown in Fig 2, the smoothing curve fitting results further revealed a positive association between the ln LAP and the chance of endometriosis incidence. Analyses showed that high ln LAP was associated with higher rates of infertility in women aged ＜35 years and those aged ≥35 years old (Fig 3). This association existed in women with a BMI ＜ 25 kg/m^2^ group (Fig 4). The smoothing curve fitting results by race are shown in Fig 5.

### Subgroup analysis

**Fig 6:**
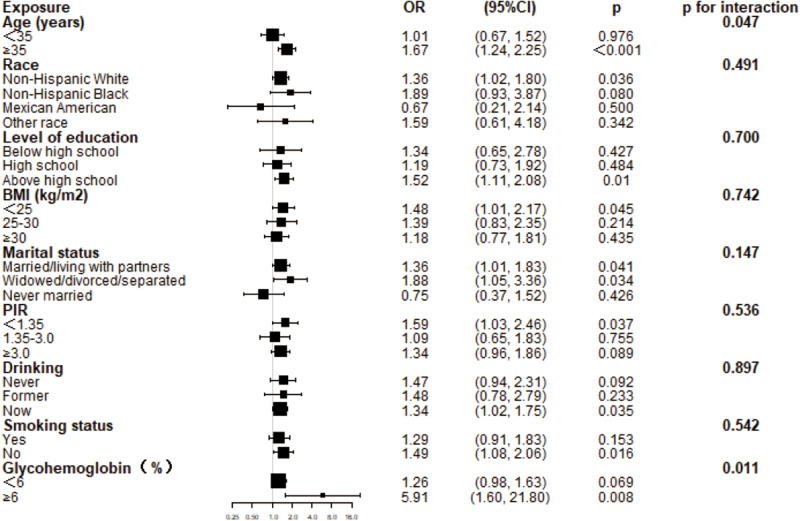
Subgroup analysis of the association between ln LAP and endometriosis. Adjust for age, race, educational level, marital status, BMI, PIR, early menstruation before the age of 11, glycohemoglobin, smoking status, and alcohol use.

Subgroup analysis and interaction tests were performed to evaluate the constancy of the relationship between ln LAP and endometriosis among factors. Stratification was based on age, race, level of education, age of menarche, glycohemoglobin, BMI, PIR, drinking, and smoking status (Fig 6).

## Discussion

This nationally representative cross-sectional research was conducted to evaluate the relationship between the LAP index and endometriosis using data from the NHANES between 1999 and 2006. It was found that ln LAP and endometriosis were positively associated among adult women in the US. According to our results, after controlling for all potential confounders, women in the highest quartile of ln LAP had a 0.93-fold increased risk of developing endometriosis compared to those in the lowest quartile. Additionally, the outcomes of the subgroup analysis proved the validity of our conclusions.

To the best of our knowledge, this is the first research that investigates the positive association between the LAP index and the odds of endometriosis prevalence, emphasizing the clinical significance of LAP in endometriosis management and prevention.

Endometriosis is a disease that causes physical and mental impact on females [26]. Thus, endometriosis is classified as a public health issue owing to its high incidence [27]. Previous studies reported that 5%-15% of women in childbearing age are affected by this disease [2–4]. In our study, the overall prevalence rate of incidence is 7.82%, which is within the prevalence range. Although endometriosis has a considerable adverse impact on medical costs and quality of life, little information is available on changes in the incidence rate of preventable endometriosis [28]. In recent years, dyslipidemia and disorders with glucose metabolism have been confirmed as one of the more significant metabolic factors of endometriosis [8–11].

Lipid accumulation product (LAP) is an indicator obtained by a combination of TG and WC (12). Research has shown that LAP is a rapid and precise index of insulin resistance, metabolic syndrome, and cardiovascular disease risks [16,29,30]. More than that, a multitude of metabolic syndromes, such as hypertension, hyperglycemia, hyperlipidemia, and abdominal obesity, are associated with the LAP index [31]. However, the relationship between the LAP index and endometriosis has not been studied yet.

In our study, logistic regression models revealed that ln LAP was positively associated with endometriosis (odds ratio 1.37, 95% confidence interval: 1.08–1.75, *P* = 0.010). After adjusting for a selection of variables, we discovered that individuals in the highest quartile of the ln LAP had a 93% higher chance of endometriosis incidence than those in the lowest quartile (odds ratio = 1.93, 95% confidence interval: 1.08–3.46, *P* = 0.027). This finding provided new insights into the association between LAP and the development of endometriosis. The LAP index may be a useful clinical tool for predicting and evaluating the incidence of endometriosis.

The possible underlying processes linking the LAP index to endometriosis are still not fully understood, and there could be some possible explanations as follows. Firstly, LAP is counted based on TG and WC values [12]. And there is growing evidence that endometriosis develops and progresses to metabolic disturbances [7–8]. Specifically, women with endometriosis have been reported to have dysregulated glucose and lipid metabolism [8]. Moreover, LAP is developed as a valuable marker that may identify American adults at cardiovascular risk more accurately than BMI [12]. Consistent with a study, there is a statistically significant association between endometriosis and an elevated incidence of cardiovascular disease [32]. Therefore, with the increase of the LAP index, the influence of the above factors may lead to further promotion of the establishment and growth of endometrial lesions.

To further investigate the relationship between LAP and endometriosis, subgroup analyses were further done. The effect of the LAP index was less obvious in women under 35 years old, but in those over 35 years, there was a positive linear relationship between the OR of LAP and endometriosis. Studies showed that the highest odds of endometriosis prevalence are in the age range of 25–35 years [33]. After the age of 35, the density of serum hormone levels, particularly the levels of estrogen, usually starts to decrease [34]. As age increases, the negative effects of estrogen gradually weaken, and abnormal metabolic conditions are important influencing factors. Thus, LAP may have significant effects on middle-aged and elderly women in its interaction with metabolic abnormalities. Interestingly, more non-Hispanic White women were self-endometriosis. The unique heredity among different races may be the reason for the different incidence of this condition in different races. Participants with endometriosis also have a lower BMI status; its underlying mechanisms are explored in further studies. Women with endometriosis had higher levels of glycohemoglobin (≥6%), which is closely related to insulin resistance. Further research could explore the potential interaction between insulin resistance and endometriosis.

Our strengths in this study are based on the NHANES population dataset, which makes results more applicable to the US population. Moreover, extensive analysis for all potential confounders and subgroups was taken in to thoroughly and carefully explore the link between the LAP index and endometriosis, which may generate more reliable conclusions. Our study is the first to provide new insights into the association between the LAP index and the chance of endometriosis occurrence. It demonstrates that LAP may play an important role in endometriosis prevalence assessment.

However, acknowledging the limitations of our study is essential. Firstly, relying entirely on NHANES participants’ self-reported diagnoses may lead to recall bias in endometriosis diagnosis. Secondly, the cross-sectional design hindered us from establishing the causal relationship between LAP and endometriosis. Thirdly, the NHANES database lacked sufficient information on the classification and severity of endometriosis, making it difficult for us to perform an analysis of ln LAP and endometriosis grading or classification. Fourthly, it is crucial to consider that NHANES might not have taken into consideration all possible confounding factors, including a family history of endometriosis, heavy menstrual periods, hormonal influences, reproductive system abnormalities, and retrograde menstruation, leading to potential bias in analytical results. Therefore, subsequent prospective studies will be conducted to validate these findings.

## Conclusion

In summary, the results of this study revealed a positive association between the LAP index and endometriosis. The findings suggested the LAP index could impact preventive measures in the future. However, it is necessary to conduct extensive and prospective investigations for subsequent studies.
